# Assessing Socket Fit Effects on Pressure and Shear at a Transtibial Residuum/Socket Interface

**DOI:** 10.1155/2023/3257059

**Published:** 2023-08-16

**Authors:** Kirstie M. Devin, Jinghua Tang, David Moser, Liudi Jiang

**Affiliations:** School of Engineering, Faculty of Engineering and Physical Sciences, University of Southampton, Southampton, SO17 1BJ, UK

## Abstract

Fluctuations in residuum volume during daily activities are known to occur in lower-limb amputees. This can cause frequent changes to fit, which cannot be accommodated by commonly-used prosthetic sockets. The real-time effects, if any, of these minor socket fit changes on interface biomechanics have not been studied extensively. Amputees commonly use different layers of socks to accommodate frequent volume fluctuations, enabling adjustment of socket fit. We, thus, altered socket fit levels via addition/removal of sock layers to a transtibial amputee who habitually-donned two-sock layers to mimic relatively looser and tighter socket fits. Interface pressure and shear sensors were placed at known prominent load-bearing sites of the transtibial residuum/socket interface, i.e., patellar tendon (PT), popliteal fossa (PF), and anterior–distal (AD) end, to measure real-time biomechanical interactions during standing and level walking. Although socket fit level was only slightly modified, changes in interface pressure and shear across anatomical sites were still observed. Tighter fit corresponds to notable pressure reduction at AD during early stance and pressure increase at PT during terminal stance due to the residuum being pushed up. Shear-to-pressure ratios were used to assess comfort, while pressure– and shear–time integrals were used to assess tissue health. We observed more notable changes at tissue sites (e.g., AD and PF). Combined evaluation of pressure and shear, including shear-to-pressure ratio and time integrals, may offer insight for residuum care.

## 1. Introduction

The lower-limb socket interface is subjected to multidirectional forces during ambulation. Impaired socket fit often leads to discomfort, injury, and reduced mobility, impeding rehabilitation outcomes [1]. Prosthetic sockets physically couple a prosthesis onto the residuum, hence its fit is critical to ensure effective load transfer, user control, comfort, and tissue safety during activities of daily living [2]. While socket fit is periodically assessed and adjusted by prosthetists in clinics, fit conditions can still frequently change due to fluctuations in residual limb volumes due to activity and weight changes [3, 4], which subsequently affect fit quality and impact tissue health. For instance, ulcers can appear in as little as 1–2 hr, especially at bony prominences [5]. In particular, as compared with transfemoral amputees, a transtibial (TT) residuum comprises many more bony prominence sites [6], and thus slight changes in socket fit level can quickly lead to localized stresses accumulating at these sites and increase risk of tissue injury [7]. TT residua volumes have also been reported to vary notably due to physical loading activities. For instance, 30 min of walking with a normal socket led up to a 6.5% residuum volume reduction and a vacuum socket resulted in up to a 3.7% residuum volume increase [8]. It is, therefore, important to evaluate interface loading caused by temporal residuum volume changes. Amputees often accommodate these changes via addition/removal of socks [9].

Sanders and Fatone [3] reported diurnal residuum fluctuations and the need for in-socket stress measurement to assess this. This challenge, especially the lack of combined in-socket pressure and shear measurements, remains an unmet need to-date hindering biomechanical understanding at this critical interface. Triaxial pressure and shear (TRIPS) sensors are thin and flexible, which are designed for loaded body interface applications, including the residuum/socket interface [10]. As a wearable sensor technology, TRIPS sensors have been successfully utilized to obtain real-time pressure and shear measurements at socket interfaces of transfemoral amputees [11, 12]. The combined pressure and shear measurements provided new insights into interface loading for transfemoral amputees. However, despite it being well known in the field that TT residua comprise more bony prominences and, thus, exhibit very different loading profiles as compared with transfemoral counterparts, there still lacks detailed studies on dynamic pressure and shear within a TT socket nor their changes with socket fit levels. This is particularly important as combined pressure and shear measurement and their ratios are important to assess comfort [13, 14] and risks to tissue viability [15].

This case study exploits TRIPS sensors, which were unobtrusively placed inside a TT socket at the anterior–distal (AD) end, patella tendon (PT), and popliteal fossa (PF) load-bearing and sensitive anatomical sites [16]. We varied socket fit by changing sock layers. By removing/adding an extra layer compared to habitually-donned two-sock fit, we aimed to simulate minor volume changes experienced in daily living. Involvement of only one TT participant enabled controlled test conditions eliminating potential differences between multiple participants; prosthesis componentry and alignment were unchanged throughout. As such, this study solely focuses on influence of socket fit. To the best of our knowledge, there are few studies on real-time pressure and shear at TT socket interfaces and lack of reports, including quantitative differences in levels of socket fit.

## 2. Materials and Methods

### 2.1. The Participant

**Figure 1:**
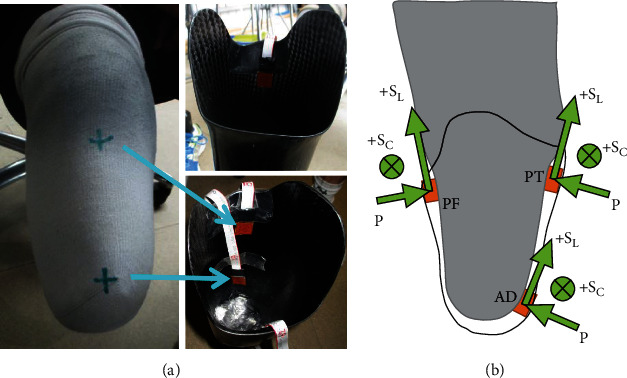
(a) Target sites marked on the amputee's residuum and in-socket sensor positions; (b) schematic indicating sensor direction definitions of pressure and shear.

One right-sided TT amputee participated (male, 38 years, bodyweight 81 kg, height 183 cm). The participant was capable of walking unassisted, and his residuum was free from injury. He used his habitual total surface-bearing socket prescribed by a certified prosthetist and achieved comfortable fit when used daily with two-sock fit and no liner (see Figure 1).

### 2.2. Instrumentation and Experimental Protocol

Upon arrival, the participant changed into Lycra shorts. Three TRIPS sensors were mounted to the inner socket wall by a prosthetist at PF, PT, and AD sites (Figure 1(a)). These are well-known anatomical load-bearing and sensitive sites for TT residua [17, 18], thus are common sites of interest in studies evaluating interface biomechanics or socket fit and comfort [7]. Both the participant and the prosthetist confirmed that there was no notable change to socket fit or comfort levels by the sensor insertions.

Each flexible TRIPS sensor has a dimension of 20 × 20 × 1 mm, which were fully calibrated with typical resolutions of 0.9 kPa for pressure and 0.2 kPa for shear measurements and further details were reported previously [11].  Figure 1(b) shows direction definitions for pressure (P), circumferential shear (+S_C_), and longitudinal shear (+S_L_).

**Figure 2:**
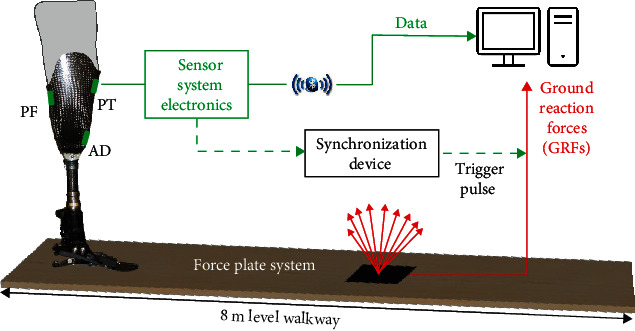
A schematic of the experimental setup.

Figure 2 shows a schematic of the experimental setup. The participant was instructed to walk for 5 min following a prescribed route to ensure there was no discomfort. A self-selected walking cadence of 117 steps per minute and speed of approximately 1.35 m s^−1^ were determined. Subsequently, level walking tests commenced, which involved a 5 s standing phase followed by walking along an 8 m level walkway at normal self-selected speed controlled using a digital metronome. A force plate (Kistler Instrument Ltd., Switzerland) was embedded halfway along the walkway to measure ground reaction forces (GRFs). At least seven clean level walking traverses were performed, and a clean traverse was defined as one with complete prosthetic foot contact with the force plate. Interface pressure and shear from the TRIPS sensor system were synchronized with GRF measurements (Figure 2) by using a 5 V trigger pulse generated by the data acquisition system of the sensor system electronics.

The participant initially donned two-sock layers, i.e., habitual condition. Subsequently, the above protocol was repeated for one-sock and three-sock layers with the fitted sensor positions unchanged. All walking tests in this study were conducted at controlled cadence (117 steps per minute) to minimize the influence of walking speeds. All tests were completed within 2 hr; the participant was able to rest between tests. Short interviews were conducted at the start and upon completion of level walking with each socket fit condition to capture feedback on socket comfort and walking stability.

### 2.3. Data Analysis

Standing baselines were used to verify that sensors were effective in measuring contact forces. GRFs obtained from force plate were used to identify key loading events during stance phase, i.e., early stance (ES, 3%–20%), midstance (MS, 20%–35%), terminal stance (TS, 35%–60%), and toe-off (60%) [19] and estimate gait cycles (GCs). Mean and standard deviation (±1 SD) were calculated for each fit condition.

Peak shear-to-pressure ratios were calculated during ES for AD and PF sites and TS for PT due to different load-dominating phases across different anatomical sites. Pressure– and shear–time integrals were also produced for comparison. Absolute sum of shear was used in the integration.

## 3. Results

**Figure 3:**
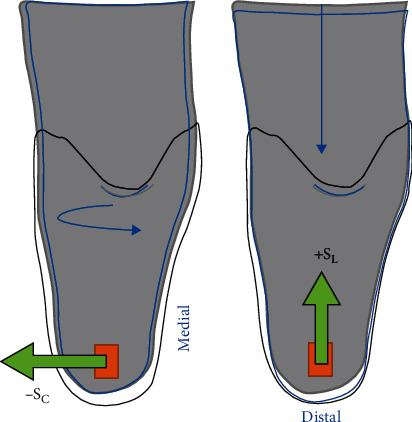
Schematic showing residuum positioning in socket based on shear directions in standing.

Table 1 displays standing baselines at the socket interface for different socks. For habitual two-sock fit, standing baseline pressure and shear at AD are greater than those obtained at PT and PF. However, pressure and S_C_ at PF and PT sites are similar. It is important to note that −S_C_ and +S_L_ are observed at the AD site simply during standing.  Figure 3 illustrates these shear directions within the socket, indicating residuum medial and downward movement, which are shown by arrows. This observation at AD site is consistent for all socket fit scenarios, as shown in Table 1.

**Figure 4:**
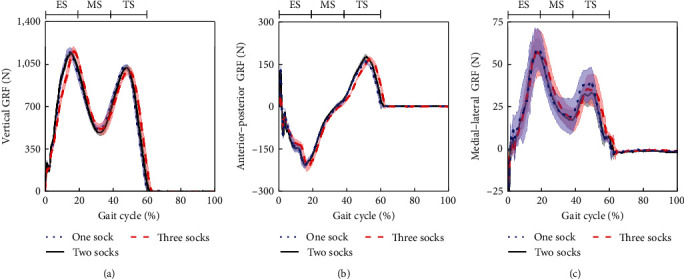
(a) Vertical, (b) anterior–posterior, and (c) medial–lateral GRF as a function of GC (*n* = 7) for different sock layers. Graphs indicate early stance (ES), midstance (MS), and terminal stance (TS) phases.

Figure 4 compares level walking GRFs for different sock layers. Vertical GRFs show characteristic gait-induced “double-hump” profiles with a peak of 1,137 ± 17 N, approximately 140% of participant bodyweight. Anterior–posterior and medial–lateral GRF of up to 208 N and 57 N, respectively, were measured. No notable changes in GRF were observed for different fits.

**Figure 5:**
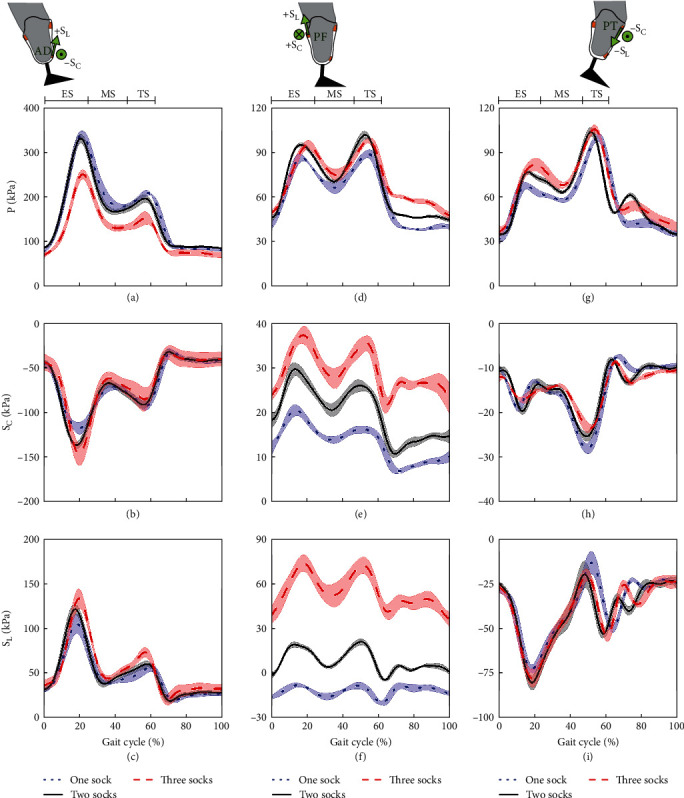
Mean and ± 1 SD of P, S_C_, and S_L_ measured at (a–c) AD, (d–f) PF, and (g–i) PT sites, respectively, as a function of GC (*n* = 7) for different sock layers during level walking. Illustrations above pressure profiles show shear directions at each site, where positive shear indicates the residuum rotates laterally (+S_C_) and moves distally (+S_L_).

Figure 5 shows pressure and shear at AD (Figure 5(a)–5(c)), PF (Figure 5(d)–5(f)), and PT (Figure 5(g)–5(i)).

As shown in Figure 5(a), at AD, higher pressure was observed during ES (250–340 kPa) than TS (152–209 kPa). In contrast, at PT (Figure 5(g)), pressure was higher in TS (99–106 kPa) than ES (68–82 kPa). Both peaks are similar in magnitude at PF (Figure 5(d)). In the second row, lateral-directional shear −S_C_ was obtained at anterior sites (Figures 5(b) and 5(h)). Like pressure profiles, relatively speaking, peak −S_C_ at AD was higher in ES than TS, whereas −S_C_ at PT was higher during TS. The third row shows proximal-directional shear +S_L_ with double-hump profiles at AD (Figure 5(c)) and PF (Figure 5(f)). However, distal-directional shear −S_L_ (up to −80 kPa) was observed at PT (Figure 5(i)).

At AD, tighter fit (i.e., three-sock fit) leads to lower pressure (Figure 5(a)) and higher shear (Figures 5(b) and 5(c)). Meanwhile, at PT, more socks tend to increase pressure (Figure 5(g)) but have little effect on shear. At the PF, different fits did not affect pressure but showed clear increase in shear S_C_ and S_L_ (Figures 5(e) and 5(f)) with increasing tightness.

**Figure 6:**
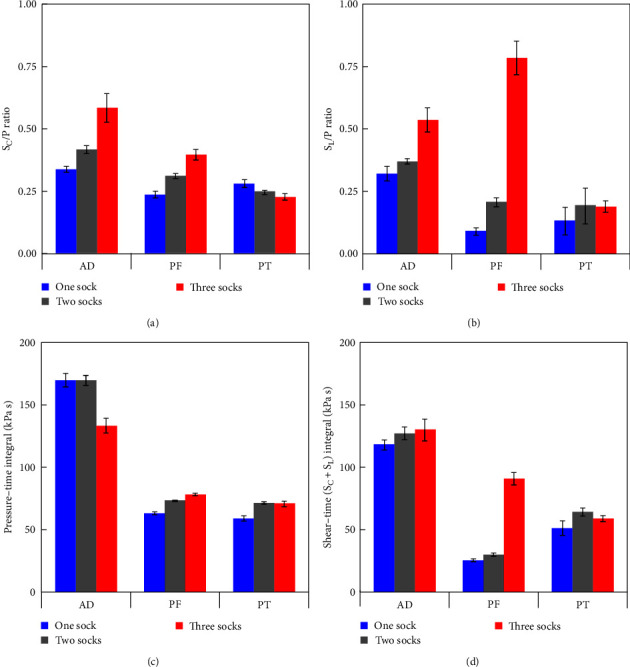
Peak shear-to-pressure ratios (a) S_C_/P and (b) S_L_/P at the point of maximum pressure and (c) pressure–time and (d) sum of shear–time integrals over GCs experienced by each site for different sock layers.

Figures 6(a) and 6(b) compare effect of different socket fits on shear-to-pressure ratios, and Figures 6(c) and 6(d) show pressure–time and shear–time integrals, respectively. Shear-to-pressure ratio, generally speaking, increased with increasing tightness. Pressure–time integral at AD decreases for tighter fit, while shear–time integral increases. Despite minor change of pressure–time integral at PF, shear–time integral shows substantial increase for tighter socket.

## 4. Discussion

Standing baselines, as shown in Table 1, depict standing loads at anatomical sites. Higher pressure (164 kPa) was obtained at AD due to the tibial-end bony prominence and the distal location. PT and PF presented lower and similar pressure of approximately 60 kPa, suggesting relatively even distribution across anterior–posterior proximal sites. Pressure distribution aligns with those in literature [17], confirming sensors were placed at load-bearing sites. Negative shear S_c_ at AD and PT combined with positive S_C_ at PF indicates the residuum rotated medially within the socket (Figure 3). Positive S_L_ shows proximal shear at AD, indicating the socket moving upward relative to the residuum. These measurements were attributable to donning whereby the residuum pushes down and rotates in the socket to achieve secure attachment. Also, in all cases, −S_C_ across anteriorly-located sites show lateral directional shear and medial directional shear at the posterior PF site, indicating the residuum has slightly rotated in the medial direction within the socket. On the other hand, +S_L_ shows proximal directional shear at AD and PF due to downward load of the residuum in socket, but opposite distal direction shear at PT, which may be caused by slight anterior tilt of the socket relative to the PT site. The cross-location profile didn't seem to change significantly with different sock layers, though pressure and shear values changed in each case. In addition, pressure at AD when using three-sock fit (127 kPa) was notably lower than AD pressure when using one- or two-sock fit conditions, which may be due to higher seating of the residuum induced by tighter fit. This briefly aligns with the participant's feedback, i.e., three-sock fit offers more support but slightly less spatial awareness of the limb with reference to the ground.

GRF profiles, as shown in Figure 4, are similar to other amputee gait studies in literature [20]. GRFs show walking patterns that were relatively unaltered when using different sock layers in our study.

In order to understand interface biomechanics, we initially analyzed results for two-sock fit, as shown in Figure 5, which was the habitual socket fit for this amputee. Figure 5 shows double-hump pressure peaks (up to 340 kPa) at all sites (Figures 5(a), 5(d), and 5(g)), indicating effective load transfer from GRFs at the measurement locations.

At AD site (Figure 5(a)–5(c)), heel strike in ES caused pressure to increase rapidly as the residuum moved down within the socket and simultaneously bodyweight shifted from contralateral to prosthetic side, leading to lateral shear −S_C_ and proximal +S_L_.

The PF (Figure 5(d)–5(f)) takes relatively even load across ES and TS, shown by balanced pressure peaks. ES pressure resulted from anterior residuum rotation in the socket, while TS pressure peak resulted from counterbalancing pressure increase at PT, aligning with dynamic anterior–posterior coupling at this interface [21, 22]. Medial +S_C_ (Figure 5(e)) and proximal +S_L_ (Figure 5(f)) at this posterior site further indicate medial rotation and downward movement of the residuum in the socket.

PT site takes more load during TS (Figure 5(g)) evidenced by higher pressure peak (99–106 kPa) than in ES (68–82 kPa). This may be due to knee flexion and the body propelling forward during TS, causing higher pressure at PT against inner socket wall. Simultaneously, the residuum rotates medially resulting in lateral −S_C_ and distal directional −S_L_ (Figures 5(h) and 5(i)).

The active knee extension, preventing knee buckling, may account for higher pressure at AD during ES than TS [16]. Presence of a natural knee allows TT amputees to retain close to natural flexion/extension mechanism ensuring limb stability during stance and safe foot clearance during swing [23]. Consequently, the distal region (i.e., AD site) is subjected to greater loading in ES as the socket rotates about the residuum [24].

Further analysis based on Figure 5 is conducted below in order to compare the effect of different socks. Increasing socket tightness, i.e., increasing sock layers, led to pressure decrease at AD (Figure 5(a)) but an increase at PT (Figure 5(g)). This indicates the residuum was slightly “pushed up” in the tighter socket case whereby AD experienced reduced pressure. Higher −S_C_ (−148 kPa) and +S_L_ (134 kPa) were also observed for tighter socket at AD, indicating the residuum trying to move to its habitual position, especially in ES. In TS, tighter fit also led to lower pressure but little change in S_C_ and S_L_.

At the PF, changing sock layers had little effect on pressure but notable impact on shear. In particular, when using three-sock fit, shear S_C_ and S_L_ were much higher compared with those of one- and two-sock fit conditions. We believe this may be due to greater tissue presence at PF, which is known to help redistribute loading more evenly compared to anterior compartments [25]. Tighter socket fit amplifies this distribution, reflected by higher shear values at the local site. In addition, a tighter fit may alter local friction coefficients at the interface, leading to higher shear. This could also be associated with the increase of static shear induced by tighter fitting.

Figure 6 shows shear-to-pressure ratio, which is a reported important criterion to assess comfort and residua tissue loading characteristics [13]. In particular, reduction of shear may improve socket comfort even if at the expense of rising pressure resulting in greater total interface stress magnitudes [3]. In our study, a tighter socket led to notable increase of S_C_/P (Figure 6(a)) and S_L_/P (Figure 6(b)) ratios at AD and PF, suggesting greater transition from pressure to shear when the socket becomes slightly tighter, which perhaps reduces the participant's perceived comfort.

Pressure–time integral is also a reported important parameter to assess cumulative exposure of pressure and time, which can lead to tissue damage, and has been considered a contributory factor in ulcer formation [26]. Unlike absolutes, pressure–time integrals consider both magnitude and time of exposure to loading, hence could offer insight into etiology of tissue damage at the residuum/socket interface. Applying this principle to shear, using pressure–time and shear–time integrals provide quantitative measures of total load exposure at each site, which is especially important when considering residua tissue viability.

For a tighter socket, we observed reduction of pressure–time integral (Figure 6(c)) but increase of shear–time integral (Figure 6(d)) at AD. This could result from pressure reduction (Figure 5(a)) at AD as the residuum was unable to move further in the socket. However, at PF, despite only minor change in pressure–time integral, there is notable change in shear–time integral, which aligns with the increase of S_C_ and S_L_ at PF, as shown in Figures 5(e) and 5(f). This suggests the tissue injury mechanism at PF is dominated by shear and its duration. Indeed, localized irritation and tissue breakdown are commonly reported [27] and believed to be associated with repetitive shear stresses. However, while many studies utilize pressure–time curves to assess tissue ulceration [28], relatively few reports focus on tissue health using shear–time integrals.

This study was limited to one TT participant as a control to test different socket fits via change of sock layers. Future work should expand to different amputees to gain populational assessment. While this study simulated changes in fit, it did not evaluate the effectiveness of altering number of socks on residuum volume fluctuations. Nevertheless, results corroborate the complex interface biomechanics which can be affected by minor socket fit changes.

## 5. Conclusions

Socket fit levels were manually altered by applying different layers of socks at the residuum/socket interface for a TT amputee. Real-time interface pressure and shear were measured and analyzed for standing and walking scenarios. We found that both circumferential and longitudinal shear existed at the socket interface during initial standing. Lateral direction and proximally-acting shear at the AD site was observed for all sock test scenarios, indicating medial rotation and downward movement of the residuum in the socket. We observed that, during walking, a tighter socket fit resulted in greater circumferential and longitudinal shear stress, particularly at areas of high tissue concentration (i.e., PF), which is subsequently reflected by high peak shear-to-pressure ratios as compared with other sites. This suggests increased axial and angular residuum movement in the socket. On the other hand, looser fit resulted in increased movement within the socket leading to distally-acting S_L_ at PT, which indicates upward local residuum movement in the socket. The results further corroborate the complex interface biomechanics, which can be affected by minor socket fit changes. This helps to demonstrate that minor changes in socket fit during daily activities may alter pressure and shear load transfer mechanisms at the interface whereby comfort and tissue integrity can be objectively assessed using these parameters.

## Figures and Tables

**Table 1:** Mean ± 1 SD of pressure and shear during standing for different sock layers.

Socket fit	Location	*P* (kPa)	*S*_C_ (kPa)	*S*_L_ (kPa)
One sock	AD	149 ± 6	−82 ± 2	56 ± 6
	PT	60 ± 3	−20 ± 5	−50 ± 4
	PF	62 ± 2	22 ± 4	0 ± 1

Two socks	AD	164 ± 9	−84 ± 6	55 ± 6
	PT	62 ± 2	−20 ± 1	−40 ± 4
	PF	64 ± 1	22 ± 4	0 ± 1

Three socks	AD	127 ± 6	−82 ± 8	70 ± 5
	PT	47 ± 2	−17 ± 2	−32 ± 2
	PF	64 ± 4	26 ± 3	69 ± 4

## Data Availability

All data supporting this study are available from the University of Southampton repository at https://doi.org/10.5258/SOTON/D2562.
